# Program manager perspectives on the service system to meet the needs of youth with concurrent disorders: findings from a Canadian national survey

**DOI:** 10.1186/s12913-015-1060-4

**Published:** 2015-09-18

**Authors:** Joanna L. Henderson, Gloria Chaim, Stephanie Luca, E. B. Brownlie, Susan Rosenkranz, Tracey A. Skilling, Joseph H. Beitchman

**Affiliations:** Child Youth and Family Services, Centre for Addiction and Mental Health, Toronto, Canada; Department of Psychiatry, University of Toronto, Toronto, Canada; Hospital for Sick Children, Toronto, Canada

**Keywords:** Concurrent disorders, Youth, Stakeholder-informed research, Knowledge exchange strategies

## Abstract

**Background:**

Concurrent mental health and substance use issues are a serious problem for adolescents and transition-aged youth. Service providers across sectors must be involved in informing system change to meet youth needs. This study examines stakeholder perspectives on services for youth with concurrent disorders including 1) clinical issues in youth services; 2) priority system issues; and 3) optimal knowledge translation strategies to enhance researcher-stakeholder communication.

**Methods:**

A database of youth clinical services across Canada was developed. Program managers (*n* = 481) at cross-sectoral (mental health, addictions, justice, child welfare, advocacy, and outreach) youth-serving (aged 12–24) programs were invited to complete an online survey; 232 responded. Survey questions concerned youth needs, program characteristics, priorities for service system enhancement; and usual and preferred knowledge translation methods.

**Results:**

Across service sectors, the mean estimated proportion of youth using services with concurrent mental health and substance use problems was 55 %. Program managers reported routine screening for mental health and substance use concerns (66 %), referring to other agencies to meet the concurrent disorder needs of youth (54 %), offering specific programming for concurrent disorders (42 %), and program evaluation (48 %). Notably, mental health programs were significantly less likely to offer concurrent disorders services than addictions programs. Where services do exist, most are targeted at youth aged 12–18 years, with fewer services available for transition-aged youth. Endorsement of various system change goals exceeded 80 %, with a particular emphasis on improving access to services (49 %), ensuring a continuum of services for varying levels of severity (37 %), and improved integration across sectors (36 %). Preferred knowledge exchange methods were workshops and websites for receiving information; and focus groups or surveys, rather than intensive participation on research teams, to inform research.

**Conclusions:**

There is a high need to build capacity across most sectors for meeting the needs of youth with co-occurring mental health and substance use problems, especially for transition-aged youth. In addition, limits in program evaluation should be addressed. Innovative knowledge exchange strategies are needed to better meet the needs of youth with concurrent disorders. Although service providers expressed readiness to participate in service enhancement and knowledge translation activities, effective, feasible approaches must integrate strategies likely to result in desired clinical outcomes, given clinical workload challenges.

## Background

Concurrent disorders, or co-occurring mental health and substance use problems, are a pressing issue for youth. In a recent study of the needs of youth presenting to cross-sectoral youth-focused services in 8 communities across Canada, 41 % of youth screened positive for both significant mental health concerns and problematic substance use [1]. Concurrent disorders are associated with impairments in functioning, behavioural problems and social marginalization [2, 3] and increased vulnerability to academic problems, employment issues, criminal involvement, and suicidal behavior [4, 5]. Youth with concurrent disorders are particularly likely to be involved in multiple service sectors, including substance use and mental health services as well as non-treatment services such as child welfare, youth justice, and housing/shelter [6], where appropriate clinical resources may be limited [7, 8]. In addition to the detrimental effects of concurrent disorders on youth and families, the cost to society is high, with billions lost both in health care costs and decreased productivity [2, 9].

Considering these costs, the provision of high quality, accessible and efficient services to meet the needs of youth with concurrent disorders is essential. While concurrent disorder treatment efficacy research is still in its infancy, emerging evidence suggests that motivational enhancement, cognitive behavioural, and family-based approaches show promise [10–13]. Unfortunately, as in other areas of health service delivery, gaps remain between research and practice [14, 15]; and it is not clear how widely or well evidence-based interventions are implemented [16], nor the extent to which services are evaluated. Moreover, current services continue to lack coordination [2, 16–18], despite the importance of effective cross-sectoral collaboration in meeting the needs of individuals involved in multiple service sectors [8, 17, 19] such as youth with concurrent disorders [6].

The complications of implementing evidence-informed practices in treatment and service delivery are often underestimated [15]. Attention is needed to the specific capacities within youth service sectors to implement system enhancements including availability and sustainability of resources; alignment with existing policies and practices; and collaborative approaches to knowledge exchange [15, 19]. Stakeholder involvement in research, practice and system change planning from the beginning of the planning process is essential to ensure relevance of new knowledge and practices; conversely, existing gaps in stakeholder involvement in enhancing understanding of clinical issues in settings where youth with concurrent disorders present for services; identifying the priority system issues to be addressed; and determining optimal knowledge translation strategies to enhance researcher-stakeholder communication are barriers to system enhancement for youth with concurrent disorders [20, 21]. Models of knowledge exchange that involve reciprocal exchanges and collaboration between researchers and stakeholders enhance the applicability and subsequent uptake of knowledge [15, 20–22].

In order to inform research and planning on system enhancement and to facilitate subsequent uptake of knowledge gained through research, the current study aimed to enhance our understanding of stakeholder perspectives on (1) clinical issues in services targeted to youth, (2) priority system issues to be addressed, and (3) usual and preferred knowledge translation strategies. To do this we developed and implemented a national online survey of program managers at cross-sectoral (mental health, addictions, justice, child welfare, advocacy, and outreach) youth-serving (age 12–24) programs. The survey gathered information about concurrent disorder-related needs of youth presenting at their programs; program characteristics, including services offered, collaboration across sectors and programs, and program evaluation activities. Three contextual factors were considered in respect to program characteristics: youth service sector (mental health, addictions, and other sectors), which may affect expertise and attention to youth with concurrent disorders [17–19]; affiliation with universities, which may facilitate integration of research and practice and has been associated with more positive attitudes toward evidence-based practice [14]; and organization size, which relates to factors such as access to resources; perceived access to resources at an organizational level has also been associated with more positive attitudes toward research and evidence-based practice [14]. In addition, the program managers were asked for their perspectives on system issues and on current knowledge translation practices, in order to inform ongoing research on youth services [14, 20–22].

## Methods

### Sample development

We developed a comprehensive list of all youth-serving programs in Canada providing addictions, mental health, child welfare, justice, advocacy or outreach services. As a first step, we consulted online service registries maintained by provincial governments. We searched for programs using the terms “addiction”, “mental health”, “child welfare”, “justice”, “advocacy”, and “outreach”, and recorded the contact information for all agencies indicating that their target population includes youth aged 12 to 24 years, or a subset of this age range. This age range was selected in order to capture services provided to both adolescents and transition-aged youth. In addition, we conducted a Google search of Canadian websites using the search terms “youth”, and “addiction or substance or mental health or justice or advocacy or outreach”. We also searched the term “child welfare”. Although some information was collected about organizations, all sampling was done at the program level. After compiling the list (*N* = 742), we called identified programs to confirm the availability of youth services and to obtain program manager (or equivalent) names and email addresses. If necessary at least three attempts were made to acquire this information. Contact information for 481 program managers (or equivalent) was obtained, and 232 surveys (48.23 %) were completed.

### Measures

As there were no existing tools that captured the information required for this study, the survey questions were adapted from a previous national survey of addictions service providers [23] and a previous survey of stakeholders [21], with additional items included specific to this study. The survey included questions about (1) *the respondent* (i.e., position, professional discipline, years of experience); (2) *the program* (i.e., service sector, youth client needs (estimated proportion with concurrent mental health and substance use concerns); types of treatment (individual, group, or family therapy; withdrawal management) and non-treatment services (academic, case management, health care, housing support, recreational programs); treatment intensity (residential; inpatient/crisis beds; day treatment; outpatient); target age range; organization size; use of concurrent disorders screening tools and program evaluation); (3) *service system* (i.e., respondent ratings and rankings of importance of addressing specific system issues); and (4) *knowledge translation and exchange* (i.e., preferred and commonly-used strategies to inform clinical practice).

### Procedure

We sent each program manager (or equivalent) a link to our anonymous online survey, along with an information page that detailed the project rationale and goals including the ultimate goal of improving services for youth and families. The information page also included information about the voluntary nature of participation in this study, confidentiality, and expected completion time (under 30 min). If necessary four email reminders were sent to encourage participation. Remuneration was not provided for participation. Participants indicated their informed consent before accessing the survey. The survey was completed during the 2012 calendar year. The study received approval from the Research Ethics Board of the Centre for Addiction and Mental Health.

### Data analysis

Because this was an exploratory study, analyses were primarily descriptive. The relationships between three contextual variables (service sector, organization size, and affiliation with an academic centre) and program characteristics were examined using *t*-test and chi square tests for continuous and categorical variables, respectively. For three-way comparisons of addictions, mental health and other, non-treatment service sectors, Tukey’s HSD post hoc comparisons were used for the continuous dependent variable; logistic regression with contrasts were used for binary dependent variables. To control Type 1 error due to multiple comparisons, α was set to .0125 for these contextual comparisons.

## Results

### Characteristics of the sample

The 232 respondents to our survey included program managers (*n* = 128), service providers (*n* = 64), executive directors (*n* = 36), and individuals in other positions (*n* = 3; 1 missing position information) from across a broad range of sectors (please see Table 1). Respondents indicated that they were from primarily small (<10 full-time equivalent (FTE) staff) programs (66.04 %), situated in primarily large (>31 FTE staff) organizations (59.81 %), serving both urban and rural geographic regions (45.79 %). In terms of affiliations with academic centres, 64 (28.19 %) reported that their organizations are affiliated with a university/college or collaborated with individuals from a university/college and 110 (48.46 %) reported that their services are not affiliated with, but are in the same community as, a university/college.

**Table 1:** Service sectors of participating programs

Sector	*n*	%
Mental health	88	37.93
Addictions	58	25.00
Child welfare	18	7.76
Justice	12	5.17
Health/primary care	7	3.02
Youth advocacy/engagement	6	2.59
Family advocacy/engagement	5	2.16
Outreach	5	2.16
Multi-service	12	5.17
Other	11	4.74
Missing	10	4.31
Total	232	100.00

For the 231 respondents who reported on the types of services provided by their programs, the most frequently offered services were assessment/consultation (*n* = 152; 65.80 %), case management (*n* = 146; 63.20 %), and individual therapy (*n* = 144; 62.34 %). Most participating programs were offered on an outpatient basis (*n* = 96; 41.56 %); however, some respondents indicated that their programs include day treatment (*n* = 35; 15.08 %); inpatient (*n* = 31; 13.36 %) or residential (*n* = 31; 13.36 %) treatment (categories not mutually exclusive; non-treatment services not included). Most respondents reported that their programs serve youth aged 12 to 15 years (*n* = 197; 84.91 %) and 16 to 18 years (*n* = 202; 87.07 %). Fewer respondents indicated serving the 18 to 21 (*n* = 122; 52.59 %) and 22 to 25 age groups (*n* = 93; 40.08 %). Across all programs, the most common mental health needs of the youth served by the participating services were anger (*n* = 179; 77.16 %), anxiety (*n* = 177; 76.29 %), behaviour issues (*n* = 174; 75.00 %), mood issues (*n* = 174; 75.00 %), and substance use problems (*n* = 173; 74.57 %).

### Program characteristics: needs of youth and screening

When asked about screening for concurrent disorder-related concerns, 154 (66.37 %) of respondents indicated that their program routinely screens for both mental health and substance use concerns; of these, 78 (50.64 % of those routinely screening and 33.62 % of all respondents) indicated that standardized screening tools for mental health and substance use problems are used by their programs. We asked respondents to estimate what proportion of youth attending their services have clinically significant problems with both mental health and substance use concerns; 175 responded. The mean estimated percent of youth with mental health and substance use concerns was 55.45 (SD = 25.01); estimates of 110 (62.9 %) respondents were 50 % or higher. Respondents’ perceptions of rates of youth concurrent disorders did not differ by treatment (addictions, mental health) sectors (M = 56.76, SD = 27.01) versus non-treatment (child welfare, youth justice, etc.) sectors (M = 53.36, SD = 28.85), nor by organization size or college/university affiliation. However, respondents from the addictions sector reported higher perceived estimated proportion of clients with concurrent disorders (M = 69.51, SD = 24.49) compared to mental health (M = 48.08, SD = 25.28, *p* = .01) and compared to other sectors (M = 53.36, SD = 28.54, *p* = .01).

### Program characteristics: concurrent disorder-related services

Fifty (29.41 %) respondents indicated that their organization has a formal policy statement regarding services for co-occurring mental health and substance use disorders. Of those reporting a formal policy, 33 (66 %) reported that the policy statement refers to providing concurrent disorder-related services within the respondents’ programs, 13 (26 %) indicated policies statements that make reference to referring youth to other services (mental health or substance use) for concurrent disorder-related services. Of all respondents, 153 (65.95 %) reported that their programs have formal or informal partnerships with other agencies regarding the needs of youth with concurrent disorders. Further, 125 (53.87 %) reported ‘often’ or ‘very often’ referring to other agencies to meet the needs of youth with concurrent disorders.

Across sectors, 98 respondents (42.24 %) indicated that their programs provide specific services to address the concurrent substance use and mental health needs of youth. Respondents from programs in the addictions, mental health and non-treatment sectors differed in the proportion offering concurrent disorder-related services (64 % vs. 46 % vs. 22 %. respectively; *χ*^2^ (2, *N* = 229) = 25.86, *p* < .001). Logistic regression predicting CD services showed that compared to non-treatment sectors, CD services were more likely to be offered in services in the addictions sector (O.R. = 7.20, *p < .*001) or mental health sector (O.R. = 3.78, *p* = .002); the latter two sectors did not differ significantly in proportion offering CD services. Size of organization and connection to a university as reported by the respondent were not related to offering concurrent disorder-related services.

Among the 98 respondents who indicated that their programs offer concurrent disorder-related services, the most common concurrent disorder-related services offered were identified as: assessment/consultation (*n* = 85; 86.73 %), individual therapy (*n* = 74; 75.51 %) and case management (*n* = 68; 69.39 %). Far fewer indicated offering recreational (*n* = 23; 23.47 %), academics (*n* = 22; 22.45 %), health (*n* = 13; 13.27 %) or housing support (*n* = 10; 10.20 %) for youth with concurrent disorders (see Table 2). In terms of intensity of services offered, 45 % reported that their programs provide outpatient concurrent disorder-related services, 15 % reported inpatient services, and 16 % reported residential treatment. Most concurrent disorder-specific services were directed to youth 12 to 18 years (12 to 15 years 81 %; 16 to 18 years 88 %), with fewer concurrent disorder-specific services for transition-aged youth (19 to 21 years: 60 %; 22 to 25 years: 44 %) (see Table 2).

**Table 2:** Services for youth with concurrent disorders (*N* = 98)

Services		*n*	%
Types of Services			
Assessment/Consultation		85	86.73
Treatment	Individual therapy	74	75.51
	Group therapy	45	45.92
	Family therapy	46	46.94
	Withdrawal management	20	20.41
Other Interventions	Case management	68	69.39
	Recreational services	23	23.47
	Academic programming	22	22.45
	Health care	13	13.27
	Housing support	10	10.20
	Other	8	8.16
Service Intensity	Outpatient	44	44.90
	Day treatment	16	16.33
	Inpatient	15	15.31
	Residential	16	16.33

Categories are not mutually exclusive and non-treatment services are not included in service intensity

### Program characteristics: evaluation activities

When program managers were asked to report about current program evaluation strategies, 113 (48.71 %) indicated that they evaluate the effectiveness of their programs using at least one of the following strategies: gathering client satisfaction information (*n* = 96; 41.38 %), collecting pre- and post-program data (25.86 %), examining administrative data (*n* = 54; 23.28 %), or conducting or participating in treatment research (*n* = 25; 10.78 %; not mutually exclusive). Use of program evaluation strategies was not related to organizational size, but was positively related to collaborating or affiliating with individuals from a university/college (*χ*^2^ (1, *N* = 232) = 6.73, *p* = .009) and to treatment (vs. non-treatment) sector *χ*^2^ (1, *N* = 208) = 10.18, *p* < .001. Respondents from the addictions sector were more likely to report program evaluation (*n* = 40; 69.00 %) than respondents from the mental health sector (*n* = 43; 47.78 %; O.R. = 2.43, *p* = .012) or respondents in non-treatment sectors (*n* = 19; 31.67 %; O.R. = 4.80, *p* < .001).

### Perspectives on system functioning and system change

When program managers were asked for their opinions about the importance of various goals for improving the system of care for youth with co-occurring mental health and substance use concerns and their families, 169 responded (see Table 3). Of these, over 80 % rated each of the following system issues as “very important”: promoting the coordination or integration of services across organizations or across service sectors, offering a continuum of services across different levels of severity, enhancing strategies for engaging children, youth, and families in services and keeping them in services, and providing continuing education/capacity building opportunities for concurrent disorders. When asked to rank order the same goals, 154 responded. The goals most likely to be ranked in the top three goals for system change were improving access to services (*n* = 75; 48.70 %), ensuring a continuum of services across different levels of severity (*n* = 57; 37.01 %), and promoting the coordination or integration of services across sectors (*n* = 56; 36.36 %).

**Table 3:** System change goals identified as ‘important’ or ‘very important’

System enhancement goal	Highly endorsed	
	*n*	%
Enhancing engagement in services	138^b^	82.63
Improving access to services	130^b^	77.84
Screening for both mental health and substance use	126^c^	75.90
Enhancing continuing engagement in services	136^b^	81.43
Ensuring continuum of services across severity levels	139^c^	83.74
Enhancing coordination across organizations	142^c^	85.54
Enhancing coordination across service sectors	139^d^	84.24
Capacity building / education on concurrent disorders	139	82.24
Implementing existing evidence-based interventions	124^a^	73.81
Developing new intervention approaches	100^c^	60.24
Evaluating interventions and which work best for specific youth and families	123^a^	73.21

Responses represent ratings of 6 or 7 on a 7-point scale with anchors “Not important” (1) to Very important (7). 169 respondents completed this question; for specific items, superscripts indicate ^a^168, ^b^167 ^c^166 and ^d^165 responses

### Knowledge translation and exchange: sources of practice-related information

Table 4 shows currently used and preferred sources of information to inform practice; 159 responded to these questions. Responses are not mutually exclusive; most respondents listed multiple strategies. Workshops or presentations and discussion with colleagues were the most commonly reported source of information currently used to inform practice. Fewer respondents reported using research literature or journal articles and very few reported using newsletters or list serves (structured broadcast email lists) to inform practice.

**Table 4:** Program knowledge exchange practices and preferences (not mutually exclusive)

Strategy	Currently used		Preferred	
	*n*	%	*n*	%
Workshops/presentations	129	81.13	117	73.58
Discussion with colleagues	122	76.73	not asked	--
Clinical literature/journal articles	99	62.26	69	43.40
Research literature/journal articles	88	55.35	63	39.62
Treatment manuals/service workbooks	75	47.17	not asked	--
Meeting with experts	71	44.65	77	48.43
Needs assessment	60	37.74	62	38.99
Program evaluations	57	35.85	not asked	--
Websites	56	35.22	99	62.26
Newsletters	20	12.58	54	33.96
Media	17	10.69	20	12.58
List serves	15	9.43	25	15.72
Social media (Facebook; blogs)	3	1.89	10	6.29
Other strategies	9	5.66	0	0.00

Responses were strategies endorsed as currently used to inform practice or as preferred strategies for knowledge gained from research to be shared with them. *N* = 159

### Knowledge translation and exchange: researcher-stakeholder communication

When participants were asked about that their preferred method for receiving research-based knowledge and evidence, 159 responded. Workshops or presentations (73.58 %) and websites (62.26 %) were identified most commonly (see Table 4). Sharing information through clinical literature/professional journals (43.40 %), research literature/journal articles (39.62 %) and ongoing access to an expert (48.43 %) were endorsed by a subset. Notably, 63 % of respondents indicated that they would like to receive practice-related information about concurrent disorders and provided their contact information for this purpose.

### Knowledge translation and exchange: researcher-stakeholder collaboration

When asked to indicate their preferences for contributing to the identification of important clinical research questions and approaches for future research projects, 147 participants responded. Of those, 41 (27.89 %) indicated that they supported using surveys; 32 (21.77 %) supported focus groups; 26 (17.69 %) supported interviews and 19 (12.93 %) supported web-based discussion forums to share their opinions. Few participants indicated that their preference was to serve on an advisory committee (*n* = 15; 10.20 %) or have full membership on a research team (*n* = 11; 7.48 %).

## Discussion

Responses from 232 program managers from a variety of youth-serving agencies and service sectors illustrate the high need for services for youth with concurrent disorders. The majority of respondents indicated that at least half of their youth clients have clinically significant mental health and substance use problems, yet a substantial proportion of programs do not offer specific services for concurrent disorders. Notably, estimated rates of concurrent disorders did not differ between treatment-focused services in the addictions and mental health sectors and non-treatment-focused services from other sectors (e.g., child welfare, housing, etc.), even though there are typically substantial differences in clinical treatment resources between these sectors [2–5]. Moreover, mental health programs were significantly less likely to offer concurrent disorders services than addictions programs, suggesting that youth who are reluctant to seek service within the addictions sector may not be able to access needed concurrent disorder-specific services. Given the importance of accessible, comprehensive services for youth in general, and for youth with high needs in particular, the lack of concurrent disorder-specific services must be addressed. These findings suggest a need to build capacity across most sectors for meeting the needs of youth with co-occurring mental health and substance use problems. Without effective services to assist youth with concurrent disorders, the impairments in functioning, emotional and behavioural problems, and social marginalization often experienced by youth with concurrent disorders [2–5] are likely to continue to occur.

Where concurrent disorder-specific services do exist, most programs targeted youth aged 12–18 years with fewer services aimed at transition-aged youth and young adults, despite increasing rates of substance abuse and concurrent disorders in this age range [7]. This lower level of reported services for transition-aged youth is consistent with previous research that has also demonstrated gaps in services for transition-aged youth and young adults [24, 25] and is of particular concern given the documented difficulties that can be experienced by youth as they transition from adolescent-focused to adult-focused services [17, 24–27]. In addition, housing and other non-clinical services for youth with concurrent disorders were less common. This area requires further exploration as holistic approaches that address not only the mental health and addictions-related needs of youth, but also the social determinants of health are considered essential for supporting the achievement of well-being in youth [17, 25–27]. In addition to limits regarding the availability of concurrent disorder services, this survey reveals limits in program evaluation. Although a number of respondents reported that they assess client satisfaction, few respondents reported using pre-post evaluation approaches or formal treatment research approaches to evaluate the effectiveness of their services. These findings suggest an opportunity to build capacity for more rigorous program evaluation strategies. Approaches that have been suggested to facilitate increased use of program evaluation strategies include enhancing motivation (communication of the importance of evaluation, buy-in across organizational levels, champions providing leadership within programs), collaboration (involvement of multiple stakeholders in defining outcomes and acknowledgment of practical challenges faced by community agencies); and capacity (technical assistance and training, use of standardized tools and tools that can be tailored to specific services or populations) [28].

Respondents endorsed several goals for system enhancement as important or very important, with particularly positive views of goals related to service coordination and integration, as well as improving children, youth and family engagement strategies. When asked to rank goals, improving access to services, access to a continuum of services, and enhancing coordination were most commonly ranked in the top three goals for system change. These findings indicate that program managers across sectors share similar concerns and priorities for system change with each other, as well as with those highlighted by various national initiatives (e.g., Mental Health Commission of Canada; National Treatment Strategy Working Group [29, 30], policy-makers [31, 32], researchers [16, 26, 33], and youth and families themselves [17, 24]. These results suggest that the timing is optimal to engage stakeholders in system change planning and activities given the degree of readiness for change expressed.

One of the important drivers of change in such systems is research; however, effectively translating research into practice is an ongoing challenge. The results of this survey are consistent with other research that demonstrates challenges in finding effective strategies for sharing knowledge about clinical approaches [33, 34]. The largest proportion of respondents reported they preferred using workshops or presentations to obtain information; however, these approaches have been shown to be of limited effectiveness in leading to the implementation of evidence-based practices [33, 34]. Notably fewer respondents indicated a preference for ongoing access to an expert, which is arguably a proxy for ongoing supervision, a strategy that is gaining momentum in academic circles following demonstrations of improvements in effective treatment implementation [33, 35].

These findings suggest a continuing need to collaborate with service providers to identify alternative and innovative approaches to sharing new practice-based knowledge. Effective, feasible approaches must take into consideration both the clinical workload challenges of service providers and the importance of achieving and maintaining the components of a new interventions that are required to gain the desired the clinical outcomes.

Studies that involve service providers from the beginning of planning, examine practices to address issues of concern to them, and evaluate those practices directly in their settings may help promote adoption and implementation [36, 37]. In the current study, more than half of respondents identified discussion with colleagues as a strategy used to inform their practice. By engaging service providers directly in the activities required to develop, evaluate and implement effective practices, we may increase the frequency with which discussions about evidence-based practice occur.

This study also suggests, however, that integration of service providers into the research process requires thoughtful attention and planning. Few respondents indicated that membership on an advisory committee or research team would be the preferred method of informing clinical research. Instead, less resource intensive approaches like surveys and focus groups were preferred by more participants. This is consistent with previous suggestions that stakeholders need a range of options for involvement in the development of new evidence-based practices, options that pay particular attention to issue of clinical workload and resourcing [21, 38]. Maintaining ongoing, respectful and responsive relationships with community agency stakeholders with opportunities for meaningful participation may enhance these collaboration efforts. Working with communities of practice, or other networks of cross-sectoral community agencies, with opportunities for capacity building that provide direct benefit to programs, may also enhance researcher-stakeholder partnerships and increase uptake of specific evidence-based practices [1].

### Limitations

This study is limited by the participation rate (48.23 %), which may reflect the heavy workloads of program managers, a lack of interest or perceived relevance of the topic, or a reluctance to participate in research. It is possible that the subset of those located who responded, by participating, may have indicated greater value placed on collaboration with research efforts; these results may therefore somewhat overestimate interest in collaboration with research efforts. Reduced response rates for questions later in the survey may also be correlated with valuing of research or with engagement with system change efforts. As well, since there is no existing database of all youth serving programs in Canada, some relevant services may not have been approached. In particular, smaller and/or more informal services with fewer resources and/or without web presence were more likely to be omitted; these may be less likely to have collaborative relationships with researchers or involved with evidence-based practice [15].

Even within sectors, and at provincial territorial levels, centralized repositories of youth program information generally did not appear to exist. In addition to the challenges created for research on youth services, this creates a communication barrier for the youth-serving sector as a whole, as well as within and across sectors. Future efforts should be made to create a repository of all youth-serving program information to improve collaboration and more importantly, for families and youth trying to identify potentially helpful services. Lack of awareness of services has been identified as a significant barrier in access to youth services [17].

## Conclusions

Many youth with concurrent mental health and substance use problems are presenting for services at programs across service sectors. The availability of services that address concurrent disorders, however, does not appear to be sufficient to meet the need. Moreover, specific gaps exist, such as in the areas of services for transition-aged youth and in services that meet the holistic needs of youth (e.g., housing, recreational programming). Program managers are interested in receiving information about practices for youth with CDs. Further, the high level of endorsement of multiple aspects of system change underscores the potential of the cross-sectoral youth services sector in Canada to participate in system-level efforts to better meet the needs of youth with concurrent disorders. There is a continuing need for innovative knowledge exchange strategies that realistically consider the capacities and resources of programs while harnessing stakeholder motivation for system change.

## Abbreviations

FTE: Full-time equivalent
